# Molecular and Cellular Interactions of Scavenger Receptor SR-F1 With Complement C1q Provide Insights Into Its Role in the Clearance of Apoptotic Cells

**DOI:** 10.3389/fimmu.2020.00544

**Published:** 2020-03-31

**Authors:** Catherine Wicker-Planquart, Samy Dufour, Pascale Tacnet-Delorme, Isabelle Bally, Yves Delneste, Philippe Frachet, Dominique Housset, Nicole M. Thielens

**Affiliations:** ^1^Université Grenoble Alpes, CNRS, CEA, IBS, Grenoble, France; ^2^CRCINA, INSERM, Université de Nantes, Université d'Angers, Angers, France; ^3^CHU Angers, Département d'Immunologie Allergologie, Angers, France

**Keywords:** scavenger receptor SR-F1, complement C1q, interaction, SPR, phagocytosis, apoptosis

## Abstract

The scavenger receptor SR-F1 binds to and mediates the internalization of a wide range of ligands, and is involved in several immunological processes. We produced recombinant SR-F1 ectodomain and fragments deleted from the last 2 or 5 C-terminal epidermal growth factor-like modules and investigated their role in the binding of acetylated low density lipoprotein (AcLDL), complement C1q, and calreticulin (CRT). C1q measured affinity was in the 100 nM range and C1q interaction occurs *via* its collagen-like region. We identified two different binding regions on SR-F1: the N-terminal moiety interacts with C1q and CRT whereas the C-terminal moiety binds AcLDL. The role of SR-F1 N-linked glycans was also tested by mutating each of the three glycosylated asparagines. The three mutants retained binding activities for both AcLDL and C1q. A stable THP-1 cell line overexpressing SR-F1 was generated and C1q was shown to bind more strongly to the surface of SR-F1 overexpressing macrophages, with C1q/SR-F1 colocalization observed in some membrane areas. We also observed a higher level of CRT internalization for THP-1 SR-F1 cells. Increasing SR-F1 negatively modulated the uptake of apoptotic cells. Indeed, THP-1 cells overexpressing SR-F1 displayed a lower phagocytic capacity as compared with mock-transfected cells, which could be partially restored by addition of C1q in the extracellular milieu. Our data shed some light on the role of SR-F1 in efferocytosis, through its capacity to bind C1q and CRT, two proteins involved in this process.

## Introduction

The class F scavenger receptor SR-F1 (1), previously named SREC-I [scavenger receptor expressed by endothelial cells (2)] or SCARF1 [as the product of the *SCARF1* gene (3)], is expressed in various cell types, such as endothelial cells (2), macrophages (4), and dendritic cells (DC) (5), and shows variable expression profiles, being most abundant in heart, placenta, lung, and spleen (6, 7).

Scavenger receptors were first defined by their ability to bind and induce metabolization of modified low density lipoproteins (LDLs) such as acetylated LDL (AcLDL) and oxidized LDL (OxLDL) (8). SR-F1 is now viewed as a multifaceted receptor (9), which can bind and mediate cell internalization of a wide range of endogenous and exogenous ligands, suggesting key roles in immune responses and tissue homeostasis.

The mature SR-F1 protein is made of a 402 amino acids extracellular domain which contains 10 epidermal growth factor (EGF)-like repeats, with seven of them containing exactly the EGF consensus sequence (CXCXXXXXGXXC), a short (24 amino acids) transmembrane domain and a relatively long (385 amino acids) cytoplasmic tail. The latter comprises a serine/proline-rich region followed by a glycine-rich region and contains numerous potential phosphorylation sites (2), strongly suggesting of a role in intracellular signaling. This long tail is only shared with another member of the class F family, a SR-F1 homolog, named SREC-II (1), or SCARF2. SREC-II is also a transmembrane protein that shares 52% identity over the extracellular domain with SR-F1 (7). Interestingly, the extracellular domain of SR-F1 engages in a heterophilic trans-interaction with SREC-II and SR-F1/SREC-II heterodimerization has been suggested to suppress the ligand binding properties of SR-F1 (7).

Alternative splicing during *SCARF1* gene expression results in multiple transcript variants (10). All membrane-bound splice variants showed receptor activity toward fluorescent AcLDL when expressed transiently in COS-1 cells (10). A SR-F1 soluble form of ~60 kDa was recently identified by Patten et al. (11) in human serum (1–20 ng/ml) and proposed to result from SR-F1 proteolysis.

SR-F1 recognizes numerous non-self molecules of invading pathogens (exogenous ligands) thus suggesting a role in host innate immunity although the fate of these interactions still needs to be clarified. SR-F1 is a receptor for bacterial proteins, including the outer membrane protein A (OmpA) from *Klebsiella pneumonia* (12) and the outer membrane porin PorB from *Neisseria gonorrhoeae* (13), for cell wall teichoic acid from *Staphylococcus aureus* (14), for β-glucan residues exposed on the cell surface of *Cryptococcus neoformans* and *Candida albicans* (3), and for hepatitis C virus non-structural protein 3 (15). SR-F1 interaction with its microbial ligands has been shown to elicit bacterial adherence to epithelial cells (13, 14) and/or to promote an inflammatory response in cooperation with toll-like receptor 2 (3, 12, 15).

Endogenous ligands for SR-F1 include heat-shock proteins Hsp70 (16, 17) and Hsp90 (18, 19). SR-F1 mediates presentation and cross-presentation of Hsp90/ovalbumin peptide complexes via the MHC-II and MHC-I molecules, respectively (18, 19). SR-F1 also binds with high affinity and internalizes the Tamm-Horsfall protein (THP), an urinary protein involved in several immunological processes (20) and the pancreatic zymogen GP2 (21), which is a close homolog of THP.

In addition, SR-F1 was shown to interact with molecules involved in the clearance of apoptotic cells, a process called efferocytosis, which is essential for the maintenance of tissue homeostasis and immune tolerance. Defects in this role are linked to the pathophysiology of autoimmune and inflammatory diseases (e.g., systemic lupus, atherosclerosis, rheumatoid arthritis) (9, 22). SR-F1 was shown to contribute to apoptotic cell removal through interaction with complement protein C1q (23). This process requires C1q binding to newly exposed phosphatidylserine (PS) on the apoptotic cell surface (24). Other proteins containing multiple EGF-like repeats in the extracellular domain, such as CED-1 (a transmembrane receptor with 16 EGF-like repeats from *Caenorhabditis elegans*) and mammalian MEGF10 (multiple EGF-like domains-10), now called SR-F2 (1), have also been reported to play a role in host defense and in the clearance of apoptotic cells (25–27) through interaction with C1q.

Of interest, we have shown previously that C1q, calreticulin (CRT) and PS interact with each other to enhance the clearance of apoptotic cells and to modulate phagocyte functions (28, 29). PS and CRT serve as C1q ligands on the apoptotic cell surface, but also interact directly with receptors of the phagocyte surface. Although initially identified as a receptor for the collagen-like regions of C1q and collectins (30), CRT was subsequently shown to bind to the globular regions of C1q (31, 32). We have confirmed the ability of CRT to interact with both the globular and collagen-like regions derived from serum C1q (28) and with a recombinant form of C1q globular regions (33). Berwin et al. (34) first reported binding of SR-F1 to CRT, a result later contradicted by Ramirez-Ortiz et al. (23) who reported negligible binding of SR-F1 to CRT. The fact that SR-F1 was involved in the uptake of apoptotic cells, together with our previous studies on CRT and C1q, prompted us to examine the molecular properties of SR-F1 in order to decipher its role during the phagocytosis of apoptotic cells by macrophages. In this study, we have revisited SR-F1 interactions with its endogenous ligands AcLDL, C1q, and CRT using its recombinant extracellular domain and we examined the role of full length SR-F1 on transfected THP-1 macrophages.

## Materials and Methods

### Proteins and Reagents

C1q was purified from human serum as described by Arlaud et al. (35). C1q-collagen-like regions (CLR) and globular regions (GR) were prepared as described previously (36). MW and A_1%, 1cm_ at 280 nm used for protein quantification were 459,300 g/mol and 6.8 for C1q, 189,900 g/mol and 2.1 for C1q-CLR, and 48,000 g/mol and 9.3 for C1q-GR. Recombinant human CRT was produced and quantified (MW = 49,431 g/mol and A_1%, 1cm_ = 16.5) as described by Païdassi et al. (28). Low density lipoprotein (LDL) was from Sigma-Aldrich and AcLDL was from Acris Antibodies. LDL and AcLDL concentrations were determined using the Quick Start Bradford 1x Dye Reagent (Biorad). The protein contents represent approximately a quarter of the total weight of the LDL samples. Alexa Fluor 568-succinimidyl ester was obtained from Molecular probes. Recombinant human CRT labeling with the succinimidyl ester conjugate was performed according to the manufacturer's protocol. N-glycosidase F (PNGase F) was purified from cultures of *Flavobacteriummeningosepticum* according to the method of Tarentino et al. (37), with the modification by Aude et al. (38).

Oligonucleotides were from Eurogentec. The list of primers used is provided in Table S1. Restriction and modification enzymes were from New England Biolabs. The pCMV6-SR-F1 plasmid was from Origene. Pfu polymerase was from Stratagene.

### Construction of the Expression Plasmids Encoding SR-F1 Extracellular Domains

#### SR-F1(1-421)

SR-F1 cDNA clone in a pcDNA3.1 vector was from Origene. We introduced a Pac I restriction site followed by a short sequence coding for 8 His residues and a stop codon just before the sequence coding for the transmembrane domain (amino acid 422): two primary PCR reactions were done, which generated overlapping DNA fragments. Using T7 and Pac R2 oligonucleotides (Table S1), a first fragment containing the sequence coding for SR-F1 extracellular domain and at the 3'end a Pac I restriction site was generated. A second overlapping fragment was synthesized using BGHRev and PacF2 oligonucleotides (Table S1). Both PCR products were combined and added to a PCR mix with T7 and BGH Rev primers. The final PCR product joined the 2 fragments into a single fragment spanning the whole region and was inserted in a pcDNA3.1 vector (with zeocin resistance) using EcoRI and XhoI restriction sites. The protein encoded by the SR-F1-pcDNA3.1 plasmid ends at amino acid 421 (Thr) of the SR-F1 protein sequence followed by 3 amino acid (Leu-Ile-Lys) and 8 His residues. All sequences were verified by DNA sequencing (GATC Biotech).

#### SR-F1(1-221) and SR-F1(1-353)

DNA fragments coding for the 221 or 353 first amino acid residues of SR-F1 were generated by PCR using T7 and either 221R or 353R oligonucleotides (Table S1) and were inserted in the SR-F1-pcDNA3.1 plasmid in place of the extracellular SR-F1 sequence using EcoRI and PacI restriction sites.

#### Site-Directed Removal of N-Glycosylation Sites in SR-F1

Codons for asparagine at the three potential N-glycosylation sites (Asn 289, 382, and 393) in human SR-F1 cDNA were replaced with those for glutamine. The expression plasmids coding for SR-F1 N289Q, N382Q, and N393Q were generated using the QuickChange II XL Site-Directed Mutagenesis Kit (Agilent Technologies), according to the manufacturer's protocol for simple mutations, using the oligonucleotides 289F and 289R, 382F, and 382R, or 393F and 393R (Table S1), and the pcDNA3.1 template containing the DNA coding sequence for SR-F1(1-421). The sequences of all constructs were checked by DNA sequencing (GATC Biotech).

### SR-F1 Production in 293-F Cells and Purification

Recombinant SR-F1 was produced in the FreeStyle 293 Expression System (Thermo Fisher). 293-F cells grown in FreeStyle 293 medium were transfected with pcDNA3.1-SR-F1 plasmids, using 293-fectin according to the manufacturer's instructions. Stable transformant cells were obtained following selection with 10 μg/ml zeocin (Thermo Fisher). The culture supernatants were harvested every 3–4 days.

In order to purify the secreted recombinant proteins, 2 ml of Ni-NTA resin (Qiagen) were added to cell culture supernatants (100 ml) and gently mixed for 3 h at 4°C. The mixture was loaded into a column, and the resin washed with 50 mM Tris-HCl pH 7.4, containing 0.3 M NaCl and 30 mM imidazole (50 ml). The protein was eluted with 5 ml elution buffer (50 mM Tris-HCl pH 7.4, containing 0.3 M NaCl and 150 mM imidazole), dialyzed against TBS (50 mM Tris-HCl, 0.15 M NaCl, pH 7.4), then concentrated to 0.2–1 mg/ml by ultrafiltration on Amicon Ultra-4 centrifuged filters (ultra cel-30K).

The molar concentration of SR-F1(20-421) was estimated using an absorption coefficient (A_1%, 1cm_) at 280 nm of 16.7 and a mass value of 49.0 kDa, as determined by mass spectrometry. A molecular mass of 47.3 kDa was used for SR-F1(20-421) N289Q, N382Q, and N393Q, by assuming the loss of 1 N-linked-glycan chain of ~1.7 kDa.

Molecular masses of 39.0 kDa for SR-F1(20-353) and 23.4 kDa for SR-F1(20-221) were used, as determined by mass spectrometry analysis. Absorption coefficients (A_1%, 1cm_) at 280 nm of 17.6 and 22.7 were used for SR-F1(20-353) and SR-F1(20-221), respectively (https://web.expasy.org/protparam/).

### Biochemical and Biophysical Characterization

Recombinant SR-F1 proteins were analyzed by sodium dodecyl sulfate-polyacrylamide gel electrophoresis (SDS-PAGE) under non-reducing conditions using Tris-HCl gels containing 10 to 14% polyacrylamide, using the method of Laemmli (39).

PNGase F treatment of purified SR-F1 proteins was performed by two successive additions of 10% (w/w) enzyme followed by incubations at 30°C for 3 h.

N-terminal sequence determination was performed using an Applied Biosystems gas-phase sequencer model 492 coupled online with an Applied Biosystems Model 140C HPLC system. Mass spectrometry analyses were performed on a Matrix Assisted Laser Desorption Ionization-Time of Flight (MALDI-TOF) mass spectrometer (Autoflex, Bruker Daltonics), operated in linear positive mode. The proteins (1 mg/ml) were diluted 1:2 to 1:10 in SA matrix [sinapinic acid (Sigma-Aldrich) 10 mg/ml in acetonitrile/water/trifluoroacetic acid (50/50/0.1 (v/v/ v)] and 2 μl were deposited directly on the target.

Circular dichroism (CD) spectra were recorded on a thermostated Jasco J-810 spectropolarimeter at 20°C using a 1 mm pathlength cuvette (Hellma France). Protein was dialyzed against a 10 mM sodium phosphate, 150 mM NaF (pH 8) buffer and used at a concentration of 8 mM. Ten spectra were acquired in the far-UV region (200–260 nm) at a scan rate of 20 nm/min.

Thermal shift assay (TSA) was performed using a real-time PCR machine (CFX Manager) and the iCycler iQ Real-Time Detection System (Bio-Rad). Two microliter of the fluorescent Sypro orange dye solution (Molecular Probes, 500 × in DMSO, diluted 5 times in water) were added to 2 μg of protein in TBS (25 μl final volume) in thin-walled 96-well PCR plates and the mixtures were heated from 20 to 100°C in 0.2°C steps. The melting temperature (T_m_), at which 50% of the protein is unfolded, is identified as the peak minimum when plotting the first derivative of the fluorescence emission as a function of temperature (–d*F*/d*T*).

### Analysis of Binding of SR-F1 and Its Variants by Surface Plasmon Resonance (SPR)

#### Interaction Measurements

Interaction analyses were performed at 25°C on a Biacore X, a Biacore 3000 or a T200 instrument (GE Healthcare). Proteins were immobilized on CM5 sensor chips (GE Healthcare) in HBS-P (0.01 M Hepes pH 7.4, 0.15 M NaCl, 0.005% (Biacore X and 3000) or 0.05% (T200) surfactant P20) using the amine coupling chemistry according to the manufacturer's instructions (GE Healthcare). Before covalent immobilization, protein ligands were diluted in 10 mM sodium acetate at the following concentrations and pH values: SR-F1(20-421) 7–40 μg/ml, pH 4.5; CRT 20–40 μg/ml, pH 4; Penta-His antibody (Qiagen) 100 μg/ml, pH 4.5; C1q 30–35 μg/ml, pH 5.5; BSA 25 μg/ml, pH 4. The immobilization levels for covalently immobilized proteins were 1,800–5,000 RU for SR-F1(20-421), 3,300 RU for CRT, 1,300–1,500 RU for Penta-His antibody, 16,000–18,300 RU for C1q, 3,000-8,600 RU for BSA. About 500 RU of SR-F1(20-421) or the deglycosylation mutants were captured on the Penta-His antibody by injecting the proteins at 40 μg/ml in the running buffer and 370 RU and 180 RU of SR-F1(20-421), SR-F1(20-353), and SR-F1(20-221) were captured on the Penta-His antibody by injecting the proteins at 16 and 160 μg/ml, respectively. A flow cell submitted to the coupling steps without immobilized protein was used as a reference for CRT. Immobilized BSA was used as the reference surface for C1q and a flow cell with immobilized Penta-His antibody was used as a blank for proteins captured through their His Tag.

Binding was measured at a flow rate of 20 μl/min (30 μl/min for CRT) in TBS buffer, pH 7.4, containing 0.005% (BIAcore X and 3000) or 0.05% (T200) surfactant P20 and 2 mM CaCl_2_ (referred to as TBS-Ca-P) or 2 mM EDTA (TBS-E-P). Analyses of LDL or AcLDL interactions were performed in the absence of surfactant. The specific binding signal was obtained by subtracting the signal from the reference surface. Regeneration of the surfaces was achieved by 10 μl injections of 1 M NaCl, 10 mM EDTA for all experiments, with the exception of proteins captured *via* the Penta-His antibody, where 10 μl injections of 10 mM glycine pH 2 were used.

#### SPR Data Evaluation

Kinetic data were analyzed by global fitting of both the association and dissociation phases for at least five concentrations simultaneously, either to a 1:1 Langmuir binding model (multiple cycle kinetics) or to a two-state reaction binding model (single cycle kinetics), using the BIA evaluation 3.2 (Biacore X and 3000) or Biacore T200 evaluation 2.0 software (GE Healthcare). Buffer blanks were subtracted from the data sets (double referencing). For the Langmuir binding model, the apparent equilibrium dissociation constants (*K*_D_) were calculated from the ratio of the dissociation and association rate constants (*k*_d_/*k*_a_). For the two-state reaction (conformational change) model, the apparent dissociation constants were calculated from the rate constants: *K*_D_ = 1/[(*k*_a1_/*k*_d1_) (1 + *k*_a2_/*k*_d2_)]. Chi^2^ values were below 5 in all cases.

### THP-1 and JurkaT Cells Culture

THP-1 and JurkaT cells were cultured in RPMI-1640 medium (Thermo Fisher). Medium was supplemented with 10% heat-inactivated fetal calf serum (FCS) (v/v), penicillin (3 U/ml), and streptomycin (3 μg/ml). All cell lines were maintained at 37°C, under a 5% CO_2_ atmosphere. THP-1 monocytes were differentiated into macrophage-like cells by treatment with 10 nM PMA (phorbol 12-myristate 13-acetate) for 48 h (40).

### Production of a THP-1 Cell Line Overexpressing SR-F1

The pCMV6-AC-SR-F1 plasmid was generated from the pCMV6-AC-SR-F1-GFP plasmid (Origene, RG207919) by inserting a stop codon at the end of SR-F1 coding sequence, using the QuickChange II XL Site-Directed Mutagenesis Kit and oligonucleotides SRF1-stopF and SRF1-stopR (Table S1). The pCMV6-AC plasmid was generated from the pCMV6-AC-GFP plasmid (Origene, PS100010) by cleavage with HindIII and PmeI (to remove the GFP coding sequence) followed by blunting and ligation with Klenow fragment of polymerase I (Thermo Fisher). The sequences of both constructs were checked by DNA sequencing (GATC Biotech).

THP-1 cells were transfected with pCMV6-SR-F1 or the empty pCMV6 vector (Mock-transfected cells) using lipofectamine LTX reagent (Invitrogen) according to the manufacturer's protocol. Transfected cells were selected with G418 (400 μg/ml), and individual clones were selected and expanded. The level of SR-F1 expression was confirmed by western blotting of cell lysates (RIPA lysis buffer, Sigma-Aldrich) under reducing conditions with a rabbit monoclonal anti-SR-F1 antibody (Abcam, ab92308) and by flow cytometry with a goat anti-SR-F1 antibody (R&D systems, AF 2409), and detected by a goat anti-rabbit IgG, HRP conjugate (Sigma-Aldrich) or a donkey anti-goat IgG, DyLight 594 labeled (Agrisera).

### Fluorescence Microscopy and Flow Cytometry Analysis

#### Immunostaining

For all experiments on THP-1 cells, FcγR blockade was performed with the FcγR-blocking solution (Miltenyi Biotec). Cells were incubated with C1q [final concentration 80 μg/ml in PBS containing 3% (w/v) BSA] on ice for 40 min and then incubated for 30 min with mouse anti-C1q Abs (A201, Quidel) diluted 1:100, and a goat anti SR-F1(R&D systems; AF 2409) diluted 1:100 followed by AF405-labeled anti-goat IgG and Cy-3-labeled anti-mouse antibodies (Jackson ImmunoResearch). FACS analyses were performed on a MACSQuant VYB Cytometer (Miltenyi Biotec) and collected data were analyzed with the MACSQuantify software. Results were expressed as MFI (median fluorescence intensity) ratio (MFI of the labeled sample/MFI of the control). For immunofluorescence imaging, cell samples were fixed for 10 min with 3% paraformaldehyde and 0.1% glutaraldehyde in PBS at 37°C. Reduction of aldehyde groups was then done by 0.1% NaBH4 in PBS, 7 min at RT before immunolabeling. Slides were mounted using DABCO solution (1,4-diazabicyclo[2,2,2]octane, 25 mg/ml; Sigma- Aldrich) in PBS/glycerol 1:9 (v/v) and were visualized under an inverted Olympus IX81 fully motorized microscope equipped with a piezo stage [Olympus and Perkin Elmer, M4D cell imaging platform, Integrated Structural Biology Grenoble (ISBG)]. Acquisition was achieved with a sCMOS camera (Hamamtsu Orca Flash4; Volocity).

#### Phagocytosis Assay and Apoptosis Induction

Mock-transfected or SR-F1 overexpressing THP-1 cells were labeled with CFSE (CellTrace™ CFSE Cell Proliferation Kit, Life Technologies) as follows : cells were washed with PBS and then resuspended at 2 × 10^6^ cells/ml in PBS-CFSE 1 μM and incubated at 37°C for 15 min. The remaining CFSE was quenched by the addition of RPMI 1640, 10% FCS for at least 10 min. THP-1 cells were then differentiated into macrophage-like cells by treatment with 10 nM PMA for 48 h at 37°C in a 24-well tissue culture plate. JurkaT cells were labeled with PKH26 dye (Sigma-Aldrich) and apoptosis was induced in culture plates (9 × 10^6^ cells in 12 ml of fresh RPMI medium) by UVB irradiation (1,000 mJ/cm^2^) at 312 nm for 9 min. Cells were then incubated for 16 h at 37°C. The apoptosis state of cells was assessed by flow cytometry using the Annexin V-FITC Kit (MiltenyiBiotec) according to the manufacturer's instructions (representative experiment shown in Figure S1).

Apoptotic JurkaT cells were then added to THP1-derived macrophages at a ratio of 5:1 (JurkaT:THP-1), without or with 80 μg/ml of C1q for 2 h at 37°C, 5% CO_2_ in serum-free RPMI. Cells were then washed and harvested with 0.25% trypsin/EDTA and immediately analyzed by flow cytometry. Phagocytosis is expressed as the percentage (%) of the CFSE- and PKH26-labeled cells in the THP-1 macrophage population. For each condition, negative controls corresponding to phagocytosis performed at 37°C in the presence of 5 μM cytochalasin D (Sigma-Aldrich) were subtracted.

#### Quantitation of Endocytosis by Flow Cytometry

Purified CRT was labeled with Alexa fluor 568 (A568) by using Alexa Fluor Protein labeling Kit (Invitrogen) according to the manufacturer's instructions. THP-1 macrophages in 12-well plates (0.5 × 10^6^ cells/well) were incubated with 0.5 ml of complete RPMI 1640 containing A568-labeled recombinant CRT at 5 and 10 μg/ml. Uptake of the protein was allowed to proceed for 30 min at 37°C and internalization was stopped by rapid washing with PBS. Cells were then harvested with 0.25% trypsin/EDTA and immediately analyzed by flow cytometry. Results are expressed as MFI ratio (MFI of experiment with CRT-A568/MFI of experiment without CRT).

#### Statistical Analysis

Significance was tested using non-parametric Wilcoxon signed-rank test for paired samples (Graph Pad Prism 5.0).

## Results

### Biochemical Characterization of SR-F1 Ectodomain

#### Production and Purification of Soluble Scavenger Receptor

**Figure 1:**
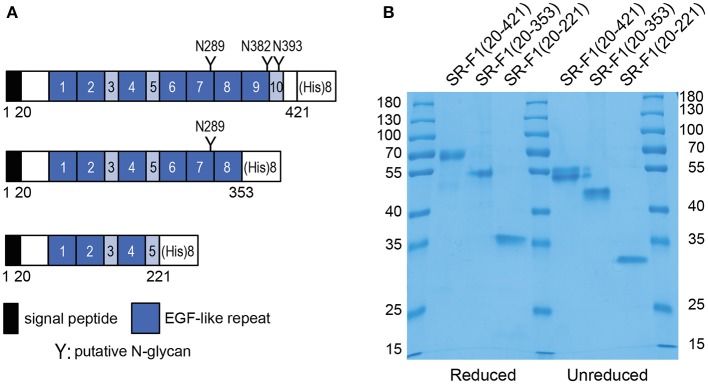
Recombinant soluble SR-F1 proteins used in this study. **(A)** Proteins (with the signal peptide) encoded by SR-F1 cDNA constructs: SR-F1(20-421), SR-F1(20,353), and SR-F1(20-221). The limits (Cys-to-Cys) of the 7 EGF modules are 41–86, 90-129, 150-190, 210-248, 252-293, 297-338, 342-381 and the limits of the other three repeats are 130-149, 191-209, 382-421. **(B)** SDS-PAGE analysis of SR-F1(20-421), SR-F1(20-353), and SR-F1(20-221) under reduced and unreduced conditions. Two micrograms of each protein were loaded on the gel (14% acrylamide). Molecular mass values of the marker are indicated in kDa.

Soluble scavenger protein constructs SR-F1(20-421), SR-F1(20-353), and SR-F(20-221), comprising EGF-like repeats 1–10, 1–8, and 1–5, respectively, were produced in 293-F cells and purified from cell culture supernatant by affinity chromatography on Ni-NTA resins. SR-F1(20-353) corresponds to alternative spliced forms of SR-F1 (isoform 4, Uniprot identifier Q14162-4 and isoform 5, Uniprot identifier Q14162-5) isolated from a peripheral blood leukocyte cDNA library (10), where EGF-like modules 9 and 10 were missing, and contains the first putative N-glycosylation site (Asn 289). To ascertain the influence of glycosylation on SR-F1 binding activities, we also produced SR-F1(20-221), which lacks EGF-like modules 6 to 10 and thus the three putative N-glycosylation sites (Figure 1A).

The mean amounts of receptor purified from 100 ml of supernatant were 450, 55, and 20 μg, for SR-F1(20-421), SR-F1(20-353), and SR-F1(20-221), respectively. SDS-PAGE analysis of the proteins revealed single protein bands for the three samples with noticeable differences in the apparent molecular weights of the unreduced and reduced protein samples (Figure 1B), which can be related to the high number of predicted cystines, i.e., 36, 30, and 18, in SR-F1(20-421), SR-F1(20-353), and SR-F1(20-221), respectively. The width of the Coomassie blue-stained protein bands observed for SR-F1(20-421) and SR-F1(20-353) probably reflected the heterogeneous glycosylation state of both proteins, in contrast to the non-glycosylated SR-F1(20-221) migrating as a thin band under non-reducing conditions.

#### Physicochemical Properties

N-terminal sequence determination of SR-F1(20-421) indicated the following sequence: SELDPKGQ, in agreement with SR-F1 sequence from residue 20. Circular dichroism was used to assess the correct folding of SR-F1(20-421). The far-UV CD spectrum of SR-F1(20-421) was typical of proteins containing EGF modules (41), displaying a negative band with a minimum at 202 nm and a weakly positive maximum around 225 nm (Figure S2A) which can be attributed to tryptophan and tyrosine residues in recombinant EGF modules (41).

SR-F1(20-421) stability was also analyzed by thermal shift assay (TSA) (Figure S2B) and a T_m_ of 46 ± 1°C was measured in TBS. Addition of NaCl up to 1 M, 2 mM CaCl_2_, or 2 mM EDTA to SR-F1(20-421) did not substantially change its T_m_ value (Figure S2B and data not shown).

### SR-F1 Is an N-Glycosylated Protein

MALDI-TOF mass spectrometry analysis of SR-F1(20-421) indicated a measured mass of 49.1 ± 0.4 kDa (average of three independent determinations), accounting for the polypeptide chain (44.0 kDa) plus an extra mass of 5 kDa putatively corresponding to N-linked glycans. Site directed mutagenesis and digestion experiments were conducted in order to confirm the presence of N-glycosylation on the three potential glycosylation sites located at asparagine residues 289, 382, and 393 (see below).

#### Receptor Deglycosylation by Peptide N-Glycosidase F Digestion

**Figure 2:**
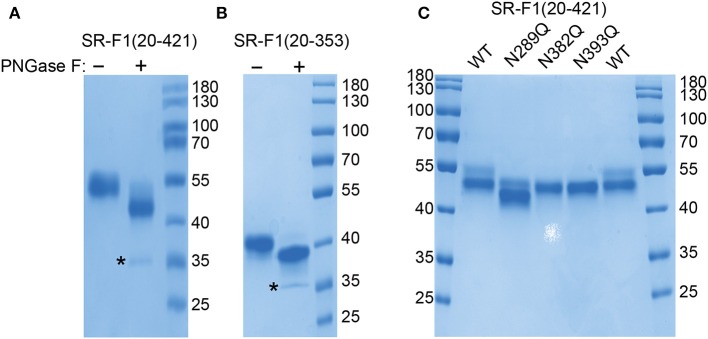
PNGase F deglycosylation of SR-F1. Digestion products of SR-F1(20-421) **(A)** and SR-F1(20-353) **(B)** were loaded on 10% **(A)** or 15% **(B)** acrylamide gel and SDS-PAGE analysis done under unreduced conditions. PNGase F (35 kDa) is indicated by a star. **(C)** Analysis of wild-type SR-F1(20-421) and SR-F1(20-421) mutants N289Q, N382Q, and N393Q by SDS-PAGE (12% acrylamide gel) under unreduced conditions. Two micrograms of each protein sample were loaded on the gels. The molecular masses (kDa) of the marker are indicated.

SR-F1(20-421) was subjected to digestion by PNGase F, which catalyzes the cleavage of asparagine-linked complex, hybrid, or high mannose oligosaccharides. Analysis of digestion products by SDS-PAGE revealed a shift in the molecular mass of PNGase F-treated SR-F1 (Figure 2A), confirming SR-F1 glycosylation. Glycosylation of N289 was directly demonstrated by the shift observed in the molecular mass of SR-F1(20-353) upon PNGase F treatment (Figure 2B).

#### Characterization of SR-F1 Mutants Lacking One Potential N-Glycosylation Site

Three SR-F1(20-421) mutants, each with one asparagine glycosylation site (N289, N382, or N393) replaced by a glutamine residue were produced and purified in the same way as wild-type SR-F1(20-421). The amounts of proteins purified from 100 ml of supernatant were 70, 85, and 200 μg, for the N289Q, N382Q, and N393Q mutants, respectively. All these mutated receptors were less secreted than their wild-type counterpart [450 μg for SR-F1(20-421) purified from 100 ml supernatant].

The purified proteins were analyzed by SDS-PAGE (Figure 2C) and MALDI-TOF mass spectrometry. The molecular masses for the N382Q and N393Q mutants were 48.0 and 47.6 kDa, respectively. The differences between the measured mass of SR-F1 mutants and the predicted mass for unglycosylated SR-F1(20-421) (44.0 kDa) are consistent with the presence of N-linked glycans on the two remaining predicted sites. This confirms that N-glycosylation is occurring on the three sites in the wild-type protein, in agreement with previous data obtained with full-length SR-F1 at the surface of Chinese hamster ovary (CHO) cells (42). PNGase F digestion of SR-F1 variants (N289Q, N382Q, and N393Q) further confirmed the N-glycan occupancy of the three putative SR-F1 sites since a similar shift of deglycosylated variants could be observed when analyzed by SDS-PAGE (Figure S3). MALDI-TOF analysis of SR-F1(20-421) N289Q yielded a molecular mass of 45.3 kDa, suggesting a possible proteolytic cleavage, which was confirmed by SDS-PAGE analysis (Figure 2C). The N-terminal sequence of this mutant was determined and was identical to that of the glycosylated receptor, suggesting that the loss of amino acids occurred in the C-terminal part of the protein. The extra loss of 2.3–2.6 kDa compared with the N382Q and N393Q mutants could be accounted for by the loss of 20–24 amino acids (considering that the average molecular weight of an amino acid is 110 Da). The absence of glycosylation at position 289 might have slightly modified SR-F1 folding, resulting in a better accessibility for proteases in the amino acids 405–409 region.

The conformation of purified SR-F1(20-421) mutants was also probed by limited tryptic and chymotryptic proteolysis where protein digestion products were analyzed by SDS-PAGE and direct Coomassie blue staining (Figures S4A,B). All mutants showed the same digestion profiles as the wild-type receptor, indicating that loss of one N-glycosylation site did not affect the receptor sensitivity to these proteases.

### Functional Analyses: Interaction of SR-F1 Ectodomain With LDL, AcLDL, and Endogenous Protein C1q

#### Interaction of the SR-F1 Ectodomain With LDL and AcLDL

**Figure 3:**
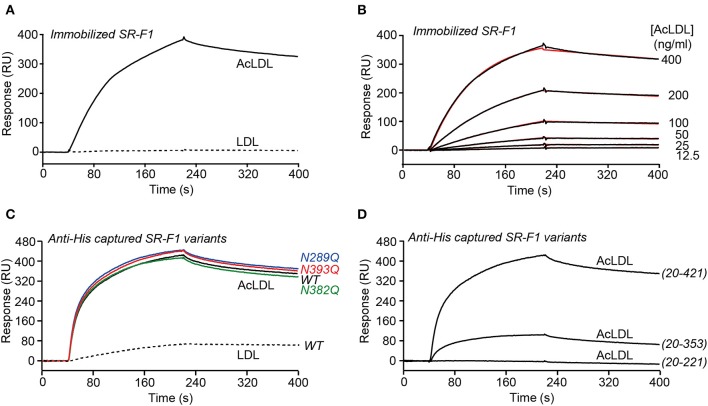
SPR analyses of the interaction of SR-F1 variants with LDL and AcLDL. LDL and AcLDL (0.4 μg/ml) **(A)** or AcLDL at the indicated concentrations **(B)** were injected over covalently immobilized SR-F1 (3,000 RU) in TBS, pH 7.4 at a flow rate of 20 μl/min. Fits (shown as red lines) were obtained by global fitting of the data using a 1:1 Langmuir binding model. **(C)** AcLDL (4 μg/ml) was injected over 450–500 RU SR-F1 and its N-deglycosylated mutants (N289Q, N382Q, and N393Q) captured on covalently immobilized anti-His tag antibody (1,300 RU). LDL (4 μg/ml) was injected over wild-type captured SR-F1. **(D)** AcLDL (4 μg/ml) was injected over equimolar amounts of captured SR-F1(20-421), SR-F1(20-353), and SR-F1(20-221) (corresponding to 520, 372, and 184 RU, respectively). Injections over oriented SR-F1 proteins **(C,D)** were performed in TBS containing 1 mM CaCl_2_ at a flow rate of 20 μl/min. The data shown are representative of at least 2 separate experiments using the T200 and the Biacore X apparatus.

SPR experiments were performed with immobilized SR-F1(20-421), either by covalent coupling to a CM5 sensor chip (Figures 3A,B) or by capture through the His-tag to covalently coupled Penta-His antibody (Figures 3C,D), using a running buffer without detergent. SR-F1(20-421) interacted with AcLDL, but not (when covalently immobilized) or faintly (when immobilized through oriented capture) with LDL, in agreement with earlier results from Adachi et al. (2) for AcLDL, and Ishi et al. (7) for LDL. The interaction with AcLDL was similar in the presence of CaCl_2_ or EDTA (data not shown), indicating that the binding was not Ca^2+^-dependent, in agreement with the absence of calcium binding consensus sequence in the EGF modules of SR-F1. AcLDL bound dose-dependently to immobilized SR-F1(20-421) (Figure 3B) with an apparent *K*_D_ value of 15.6 ng/ml, corresponding to ~5 pM, LDL reported molecular mass varying between 2.4 × 10^6^ to 2.5 × 10^6^ Da (43–45).

The ability of partially deglycosylated SR-F1 mutants to bind AcLDL was also tested. The three deglycosylated SR-F1 mutants bound to AcLDL similarly to wild-type SR-F1 (Figure 3C). On the other hand, we also observed a drastic reduction in the binding of SR-F1 (20-353) fragment to AcLDL, which represents 23% of that of entire ectodomain, whereas SR-F1(20-221) fragment did not bind AcLDL (Figure 3D). Altogether, these data demonstrated that the C-terminal moiety of SR-F1 ectodomain was strongly involved in the interaction with AcLDL.

#### Interaction of SR-F1 Ectodomain With Complement Protein C1q

**Figure 4:**
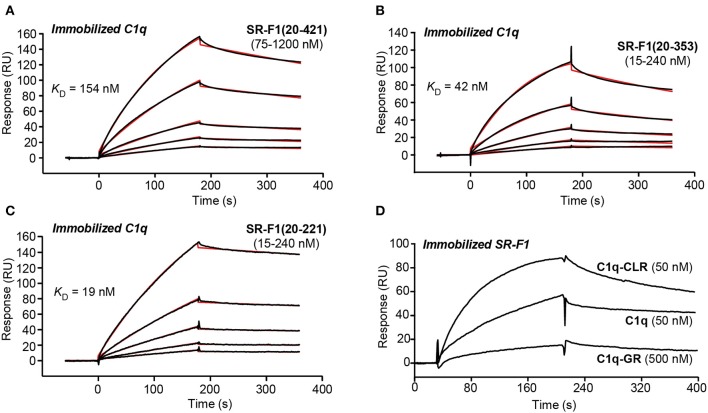
SPR analyses of the interaction of SR-F1 variants with C1q. SR-F1(20-421) **(A)**, SR-F1(20-353) **(B)** and SR-F1(20-221) **(C)** were serially diluted and injected at five increasing concentrations in multiple cycle kinetics mode over covalently immobilized C1q (about 16,000 RU) in TBS-Ca-P at a flow rate of 20 μl/min. Fits (shown as red lines) and apparent *K*_D_ values were obtained by global fitting of the data using a 1:1 Langmuir binding model. **(D)** C1q, its collagen-like region (C1q-CLR) and its globular region (C1q-GR) were injected at the indicated concentrations over covalently immobilized SR-F1(20-421) (5,800 RU) in TBS-Ca-P at a flow rate of 20 μl/min. The data shown are representative of 3 **(A**–**C)** and 2 **(D)** separate experiments on different surfaces.

SPR experiments showed that SR-F1(20-421) readily interacted with immobilized C1q with a *K*_D_ value of 186 ± 21 nM (Figure 4A, Table 1), in line with the affinity of 124 nM reported for the interaction of extracellular SR-F1 and immobilized C1q (23). Similar experiments were performed using SR-F1(20-353) and SR-F1(20-221) fragments (Figures 4B,C). Interestingly, the 20-221 and 20-353 SR-F1 fragments not only retained the ability of SRF-1(20-421) to bind C1q, but also interacted with a better affinity: 32.4 ± 6.4 nM for SR-F1(20-353) and 17.7 ± 0.9 nM for SR-F1(20-221) (Table 1). These results indicate that C1q binds to the N-terminal moiety (aa 20–221) of SR-F1 and that loss of the C-terminal part of SR-F1 seems to enhance C1q interaction with the receptor.

**Table 1:** Kinetic and dissociation constants for binding of SR-F1 to immobilized C1q.

**SR-F1 variant**	***k*_**a**_ (M^**−1**^ s^**−1**^)**	***k*_**d**_ (s^**−1**^)^**a**^**	***K*_**D**_ (nM)^**a**^**	***t*_**1/2**_ (s)^**b**^**
(20-421)	6.84 ± 0.99 × 10^3^	1.26 ± 0.20 × 10^−3^	186 ± 21	550
(20-353)	7.00 ± 2.41 × 10^4^	2.09 ± 0.44 × 10^−3^	32.4 ± 6.4	331
(20-221)	1.85 ± 0.39 × 10^5^	3.03 ± 0.74 × 10^−3^	17.7 ± 0.9	229

a *Values are the means ± SE of three experiments on different surfaces*.

b *Half-lives of complexes were determined from the k_d_ values using the formula t_1/2_ = ln2/k_d_*.

We also examined the functional relevance of the three N-glycans attached to SR-F1 by characterizing SR-F1 mutants lacking each of the N-glycosylation sites. All mutants were able to bind C1q (Figures S5A–C) with comparable *K*_D_ values around 600 nM (581 ± 9 nM for SR-F1 N289Q, 604 ± 6 9 nM for SR-F1 N382Q and 589 ± 20 nM for SR-F1 N393Q, *n* = 2), reflecting a slightly lower apparent affinity of the SR-F1 variants for C1q than wild-type SR-F1.

Finally, to further locate the SR-F1 binding site on C1q, we used the two functional regions of C1q: C1q-CLR and C1q-GR. C1q is a large molecule (460 kDa) of 18 polypeptide chains assembled into 6 triple helices of A-chain, B-chain, and C-chain, each surmounted by a heterotrimeric globular head. C1q collagenase digestion allows preparation of its GR (~48 kDa), each being a trimer of C-terminal gC1q modules (about 135 residues of one A-, one B-, and one C-chain), and being involved in the binding of a wide range of ligands (46). C1q-CLR (190 kDa) is composed of the N-terminal collagen-like sequences of the 18 polypeptide chains and interacts with proenzymes C1r and C1s. In our SPR experiments, we observed that immobilized SR-F1 binds C1q-CLR, whereas no or very weak binding was observed for C1q-GR (Figure 4D). The higher response of C1q-CLR when compared to that of whole C1q may be explained by the enhanced accessibility of the collagen tail to SR-F1.

### The Expression of SR-F1 Modulates the Efferocytosis Capacity of Macrophages

**Figure 5:**
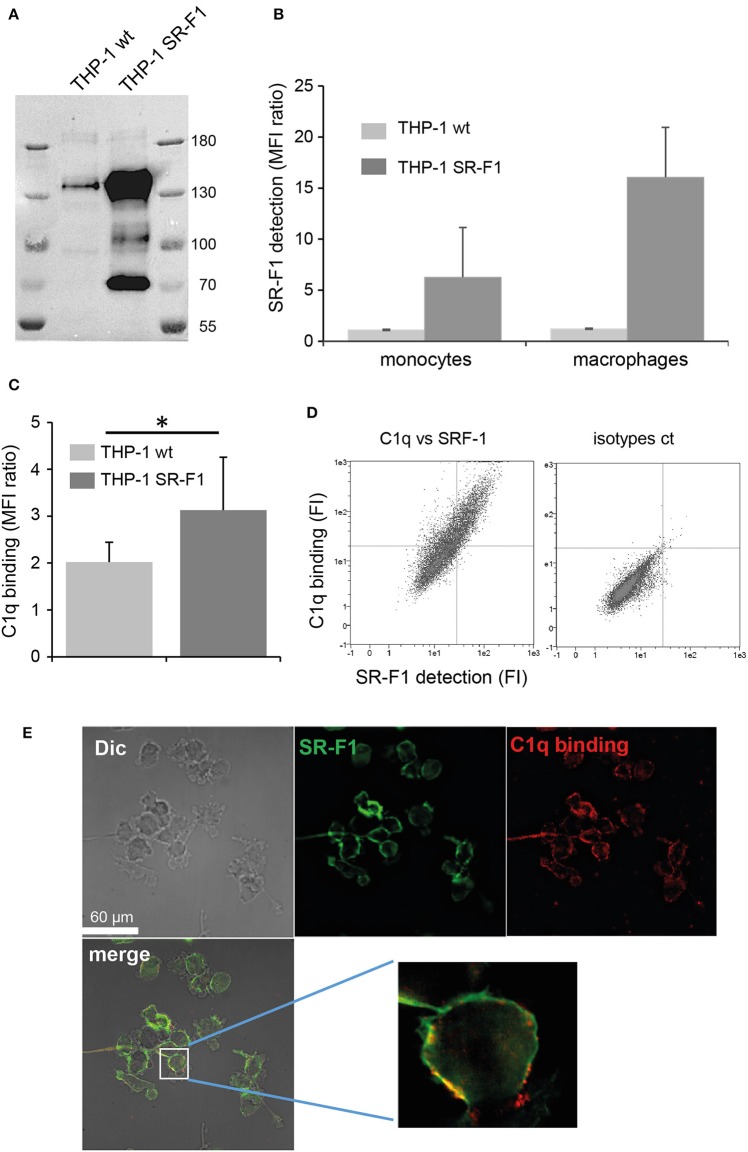
SR-F1 overexpression increases C1q binding to macrophages. **(A)** SR-F1 detected by western blotting on wild-type or SR-F1 overexpressing THP-1 cells (PMA-treated), following SDS-PAGE on a 7.5% acrylamide gel under reduced conditions. Mass markers (kDa) are shown. **(B)** SR-F1 cell surface expression measured by FACS (*n* = 3) on THP-1 cells differentiated or not into macrophages as indicated. **(C)** C1q binding to the THP-1 cells (PMA-treated), analyzed by FACS. **(D)** A representative dot plot of the FACS analysis of C1q binding vs. SR-F1 expression (left) and the corresponding control (right). **(E)** C1q and SR-F1 colocalize on the surface of SR-F1 overexpressing THP1 macrophages. Samples were visualized by confocal microscopy under differential interference contrast (DIC). AF 405 (shown in green) and Cy-3 (shown in red) filters, and merge are shown (as indicated). Higher magnification is shown for one selected cell. MFI, median fluorescent intensity. *p < 0.05. Experimental conditions are described in Materials and Methods.

In order to investigate the potential role of SR-F1 on the C1q-dependent modulation of the clearance of apoptotic cells, monocyte THP-1 cells were transfected with full length SR-F1 cDNA and a stable SR-F1 overexpressing THP-1 cell line was generated as described in Materials and Methods. These cells expressed a higher level of SR-F1 than wild type THP-1 cells, as demonstrated by western blot analysis (Figure 5A). In addition, as shown by flow cytometry analysis in Figure 5B, the SR-F1 receptor was efficiently exposed at the surface of the cell. Of note, the ectopic expression of SR-F1 increased on PMA- treated THP-1 cells, which might result from the known PMA-mediated activation of the CMV promoter (47).

Further experiments were then conducted to analyze the consequences of SR-F1 overexpression on C1q binding. As shown in Figure 5C, C1q bound more strongly to the surface of SR-F1 overexpressing macrophages. Binding was dependent on the amount of surface SR-F1 as double C1q/SR-F1 labeling experiments, analyzed by flow cytometry, showed that the more THP-1 cells expressed SR-F1, the more they were recognized by C1q (Figure 5D). We then examined the possible colocalization of SR-F1 and C1q at the surface of the macrophage using the new SR-F1 overexpressing THP-1 cell line. As shown in Figure 5E, C1q and SR-F1 colocalized in some membrane areas (yellow in the merge view), suggesting their possible direct interaction at the surface of the cell. Further supporting this direct interaction, recombinant SR-F1(20-421), when added to soluble C1q, was able to partly inhibit C1q binding to SR-F1 overexpressing cells (flow cytometry analysis shown in Figure S6).

**Figure 6:**
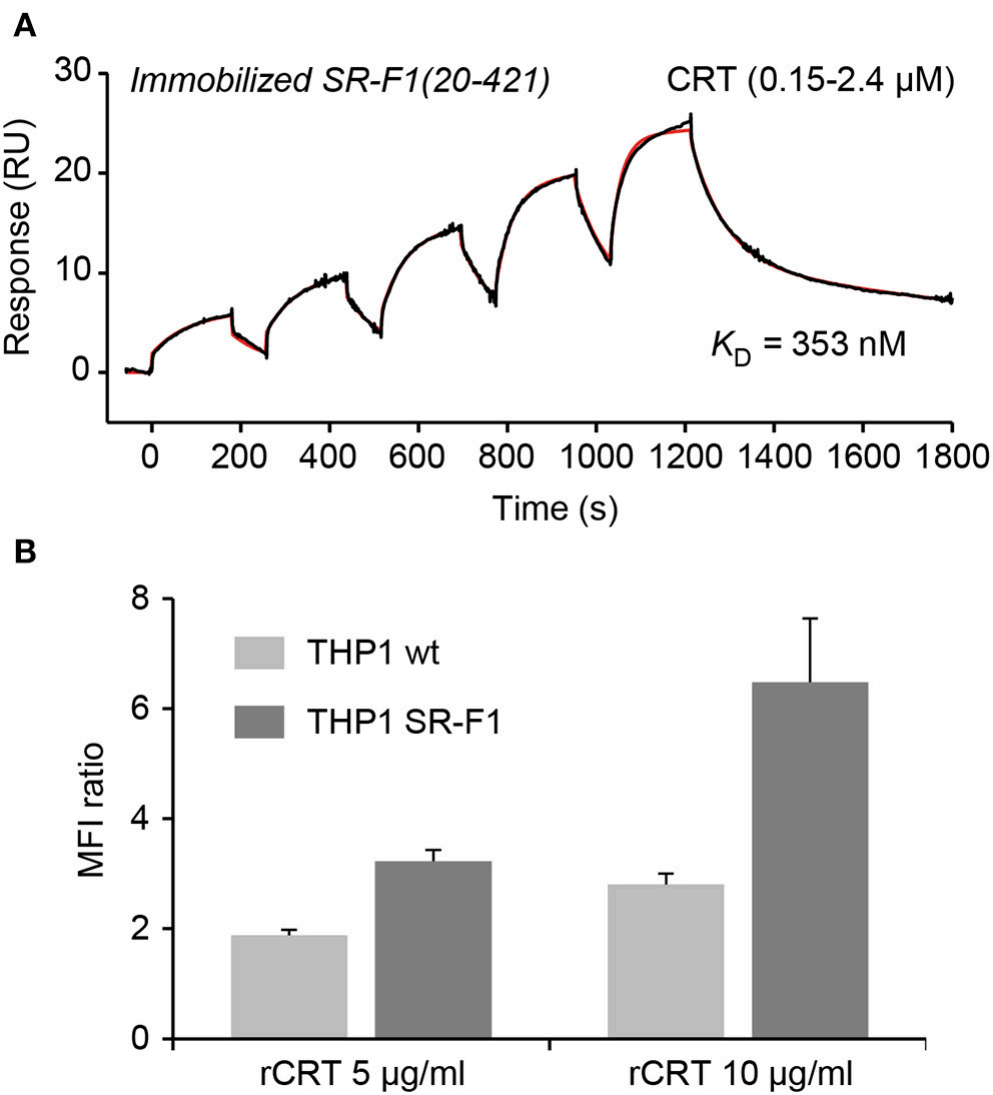
SR-F1 interacts with CRT and SR-F1 overexpression modulates the endocytosis of CRT. **(A)** CRT was serially diluted and injected at five increasing concentrations in single cycle kinetics mode over covalently immobilized SR-F1(20-421) (1,845 RU) in TBS-Ca-P at a flow rate of 30 μl/min. The fit (shown by a red line) and the apparent *K*_D_ value were obtained by global fitting of the data using a two-state reaction model. The curves shown are representative of two independent experiments. **(B)** Quantification of the endocytosis of soluble recombinant CRT (AF568-rCRT) by flow cytometry (MFI ratio ± SD, *n* = 3).

As SR-F1 has been reported to trigger the endocytosis of CRT (34) and in view of our previous demonstration that soluble CRT, released from apoptotic cells, was effectively endocytosed by macrophages (40), we analyzed the direct molecular SR-F1/CRT interaction. SPR analysis showed that CRT bound to the immobilized ectodomain of SR-F1 with apparent *K*_D_ values of 345 ± 8 nM (Figure 6A). The interaction was also observed in the reverse configuration using immobilized CRT (3,320 RU) and soluble SR-F1(20-421) and an apparent *K*_D_ of 770 nM was obtained (not shown). Interestingly, SR-F1(20-353) and SR-F1(20-221) fragments retained a similar capacity to interact with CRT, suggesting that the CRT binding site is located in the 20-221 SR-F1 fragment (Figure S7). We then investigated the efficiency of THP-1 macrophages in CRT endocytosis and observed a higher level of CRT internalization for SR-F1 overexpressing THP1 cells (Figure 6B). Our results are in agreement with those of Berwin et al. (34) obtained using a CHO cell line expressing the SR-F1 receptor. Together, these data strongly support the fact that the SR-F1 receptor is expressed by THP-1 cells in an active form.

**Figure 7:**
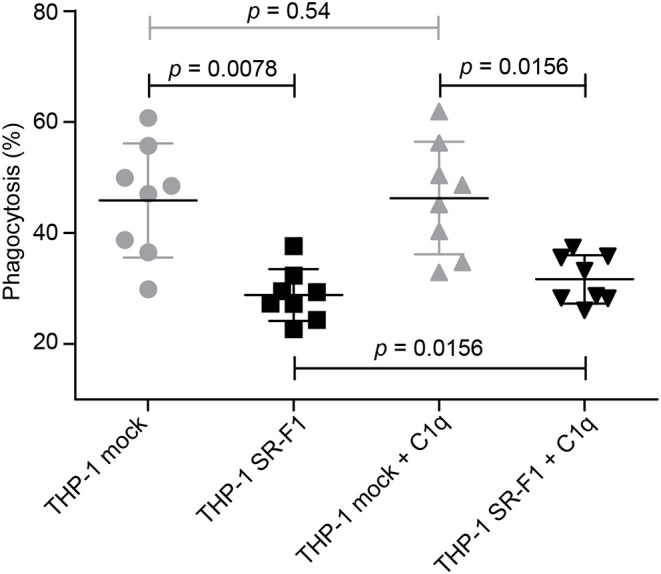
Comparative phagocytosis of late apoptotic JurkaT cells by THP-1 and SR-F1 overexpressing THP-1 macrophages, in the presence or absence of C1q. The scatter plot shows (*n* = 8) a horizontal line at the mean ± SD. Exact *p*-values are indicated. Experimental conditions are described in Materials and Methods.

We next investigated the effect of SR-F1 overexpression on the phagocytosis of an apoptotic cell population by comparison with mock-transfected cells. THP-1 macrophages were fed for 2 h with late apoptotic JurkaT cells obtained by UVB irradiation (>85% Annexin V positive and >60% Annexin V positive and PI positive, Figure S1). The uptake of apoptotic cells by THP-1 macrophages was analyzed in the absence or presence of C1q used at a physiological concentration (80 μg/ml) (Figure 7). Flow cytometry data showed that THP-1 cells that overexpressed SR-F1 presented a 1.6 fold significant decrease in the phagocytosis efficiency compared to the mock-transfected cells (phagocytosis of 28.8% vs. 45.9%, *n* = 8). This observation suggests that increasing cell surface SR-F1 could negatively modulate the function of other molecules involved in the engulfment of apoptotic cells. Interestingly, we observed a slight but statistically significant (31.7% vs. 28.8%, *p* = 0.0156, *n* = 8) increase due to the addition of C1q in the phagocytic milieu of the SR-F1 overexpressing THP-1 macrophages. This is consistent with the putative bridging function of C1q.

## Discussion

The purpose of this work was to investigate the interaction of scavenger receptor SR-F1 with three different partners, AcLDL, C1q, and CRT, and to clarify the functional fate of these interactions at the cellular level. The present work first provides a clear picture of the interactions underlying the bridging role of C1q. The N-terminal half of the ectodomain of SR-F1 is interacting with the collagen tail of C1q, whereas the globular heads of C1q bind phosphatidylserine, DNA or CRT. In this configuration, SR-F1 holds C1q quite tightly through its collagen tail and the six globular heads of C1q can capture the apoptotic cell, thanks to its avidity for several “eat me” signal molecules at the surface of apoptotic cells. The half-life of the SR-F1-C1q complex as derived from the *k*_d_ value is about 10 min (Table 1), and the half-life of C1q/CRT complex is about 16 min (28), values compatible with the duration of cell-cell interactions and the phagocytosis process. Thus, the kinetics parameters of SR-F1 binding argue in favor of a genuine capture of C1q by SR-F1. In addition, SR-F1 was able to interact with ficolins, other complement recognition proteins with collagen-like regions (our unpublished observations), which suggests that this receptor might be shared with other innate immune proteins of the defense collagens family (collectins, ficolins). These observations add SR-F1 to the list of C1q/collectin receptors for collagen-like regions, including leukocyte-associated immunoglobulin-like receptor 1 (LAIR-1), α2-β1 integrin, CD91 or complement receptor 1 [reviewed in (48)]. Further studies will be needed to identify the precise binding sites in C1q CLR and in SR-F1 EGF-like modules.

Our results identify two different binding regions on SR-F1: one is located in the N-terminal end that contains the first 5 EGF like modules of SR-F1 and includes C1q and CRT binding site(s). The second one is located in the C-terminal end that contains the 5 last EGF like modules and includes the AcLDL binding site. The SR-F1 binding site that is the more distal from the membrane is dedicated to interaction with molecules involved in the uptake of apoptotic cells. On the other hand, the binding site closer to the membrane is involved in the internalization of protein complexes. This thus suggests that the two regions may have different functions, although the binding of more partners such as hsp70 or hsp90 needs to be investigated to test the general character of this hypothesis.

Our data also suggest that the binding properties of SR-F1 for either AcLDL or C1q do not strongly depend on the presence of glycosylation on asparagine 289, 382, or 393. The mutation of any of these asparagines to glutamines does not impact at all the interaction with AcLDL, whereas the affinity of SR-F1 mutants for C1q is only decreased by a factor 3. These findings are not in full accordance with the data of Sano et al. (42) who reported that mutations of N382 and N393 increased the binding of fluorescent AcLDL (DiI-AcLDL) to CHO cells transfected with SR-F1 variants. However, cell surface SR-F1 quantification is not as accurate as that of SR-F1 immobilized at the sensor chip surface and reported affinities by these authors only differed by a factor 2 (mean *K*_D_ values of 0.35 and 0.37 μg/ml for N382Q and N393Q cells, respectively and of 0.72 and 0.66 μg/ml for wild type and N289Q cells, respectively).

The data presented in this work suggest that a change in the SR-F1 conformation is induced upon C1q binding. Indeed, the higher affinity of C1q for the SR-F1(20-353) and SR-F1(20-221) constructs, in comparison to the full ectodomain (20-421) can be explained by a conformational change of the latter upon C1q binding. A more detailed analysis further supports this hypothesis. The similar dissociation rates (*k*_d_) observed for all three constructs (Table 1) support the idea of a conserved molecular interface between any of the SR-F1 constructs and C1q. The higher association rates observed for the 20–221 and the 20–353 constructs (25 times and 10 times higher *k*_a_, respectively) suggest that the access to the binding site is facilitated. In its native state, SR-F1 may not be fully elongated but most likely folded, with the C-terminal end partially masking C1q and CRT binding site in the N-terminal half on the SR-F1 ectodomain.

Two of the SR-F1 constructs studied in this work (20-421) and (20-353) correspond to soluble SR-F1 fragment expressed *in vivo* (10, 11). The expression of soluble forms of SR-F1 may be a mechanism to antagonize the role of the membrane-bound form of SR-F1. The reduced AcLDL binding capacity of the SR-F1(20-353) construct suggests that different soluble isoforms may selectively antagonize either only the interaction with C1q or CRT (20-353) or interactions with all types of partners (20-421). Thus, the functions of the membrane bound SR-F1 may be selectively regulated by the production of one of these soluble forms.

Another goal of our study was to examine the effect of SR-F1 on efferocytosis. We showed that C1q binds more efficiently to THP-1 macrophages that overexpress SR-F1, than to wild-type THP-1 cells. This is consistent with a direct interaction between C1q and SR-F1 at the surface of the cell. The observation of colocalizations between C1q and SR-F1 after immunolabeling supports this idea and is in line with the published data from Ramirez-Ortiz et al. (23) which demonstrated that C1q binds to SR-F1-TNF-R1 chimeric receptor expressed on HEK293T cells. However, the fact that our colocalization experiment shows that C1q does not exclusively colocalize with SR-F1 strongly suggests that C1q also binds to other receptors at the THP-1 macrophage surface, such as CD91, LAIR-1, and CD33 (49–51). In the same study, Ramirez-Ortiz et al. (23) also demonstrated that SR-F1 mediates apoptotic cell recognition and capture by CD8α+ DC subset in mice from the comparison of *SCARF1*^−/−^ and *SCARF1*^+/+^ DC. It is nevertheless important to note that, in apparent contradiction with the above observation, our phagocytosis assays showed that increasing SR-F1 on THP-1 macrophages negatively modulates the uptake of apoptotic cells. This difference may be attributed to differences in the cellular model (human macrophages instead of mouse DC) or in the experimental setup. Indeed, because wild type THP-1 cells express endogenous SR-F1, it can be speculated that increasing cell production of SR-F1 molecules could affect the equilibrium in molecular interactions that control the binding and the uptake of the phagocyte's target. Thus, this could suggest that SR-F1 interferes with other molecules crucial for this process. CRT, which is known as an “eat-me signal” when exposed at the surface of apoptotic cells and is able to bind to macrophage's surface, is thus one possible candidate. To support this hypothesis, we demonstrated direct CRT-SR-F1 interaction by SPR experiments (Figure 6A) and, in line with this data, we observed that cells overexpressing SR-F1 increase their endocytic capacity for exogenous CRT, in agreement with previously published data using a mouse macrophage cell line overexpressing SR-F1 (34). This observation may explain the decrease in phagocytic efficiency. Indeed, increasing SR-F1 at the macrophage surface will increase endocytosis of CRT, regardless of whether this CRT was present at the apoptotic cell surface, or in solution. A possible consequence may be to diminish CRT availability at the apoptotic cell-phagocyte interface and consequently reduce its ability to trigger “eat-me signaling.” A possible complementary explanation for the decrease of phagocytic efficiency is provided by our previous demonstration (40) that exogenous CRT, added to wild type THP-1 macrophages and endocytosed, triggers a peculiar polarization and affects engulfment of apoptotic cells. A third hypothesis might be a possible increase of the proteolytic release of soluble SR-F1 from the cell surface, a phenomenon recently described by Patten et al. (11). Soluble SR-F1 shed from the surface of SR-F1 overexpressing cells might compete with C1q and CRT for binding to cell surface SR-F1. Alternatively, it could also be proposed that a higher concentration of apoptotic cells could be required to activate SR-F1-dependent phagocytosis in cells that overexpress SR-F1 compared to wild type macrophages. Although apoptotic cells were added in excess to THP-1 macrophages and were not all engulfed, we cannot exclude this possibility.

Given that CRT is known to bind to C1q with slightly higher affinity (*K*_D_ ~120 nM) (28) than to SR-F1 (*K*_D_ ~345 nM), we might also consider a role for possible multimolecular interactions involving C1q, CRT, and SR-F1 at the surface of the macrophage. Although CRT and C1q bind to the same half of the SR-F1 ectodomain, their respective exact binding site remains to be determined. Such an information is required to assess the possible intermolecular interaction network involving these three molecular partners.

**Figure 8:**
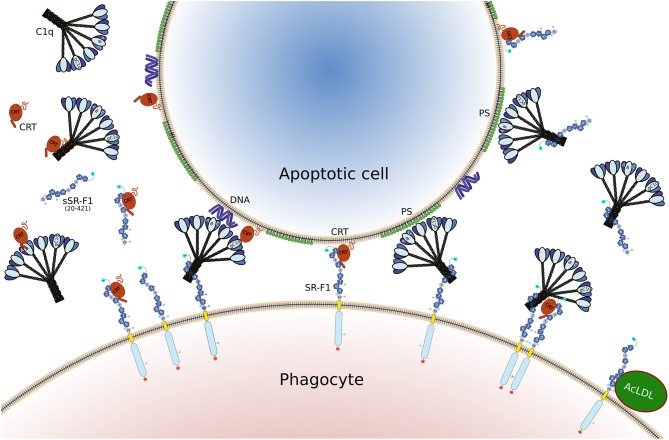
Illustration of the different interactions involving SR-F1, C1q, CRT, and AcLDL, as discussed in the present article. The full length SR-F1 includes 7 EGF like domains (dark blue pentagons numbered 1, 2, 4, 6–9), 3 EGF repeats containing domains (small medium blue pentagons numbered 3, 5, 10), a transmembrane domain (yellow) and a cytosolic domain (light blue). The N- and C-terminal extremities of SR-F1 are indicated by a cyan and red circle, respectively, and the N-linked glycans by a circled N. Soluble forms of SR-F1 extracellular domain (sSR-F1) are also shown but for the sake of simplicity only the soluble SR-F1(20-421) form has been depicted. In the center, possible phagocyte-apoptotic cell interactions mediated by SR-F1 are shown. On the left and on the right, molecular interactions interfering with the phagocyte-apoptotic cell interaction involving soluble CRT and sSR-F1, respectively. On the lower right, the SR-F1-AcLDL complex is shown as well as a putative hetero-tetramer in which soluble CRT may enhance anchoring of C1q to SR-F1 and SR-F1 clustering with possible consequences on phagocyte-apoptotic cell interactions.

Overall, the data presented here shed some light into the role of SR-F1 in efferocytosis, through its capacity to bind C1q and CRT, two proteins known to participate in the clearance of apoptotic cells, as illustrated schematically in Figure 8. It is clear that these interactions are mediated by a region of SR-F1 not involved in its typical scavenging function for modified LDL, but further experiments are needed to elucidate their functional consequences in different cellular environments. SR-F1 interacts with C1q, a bridging molecule facilitating apoptotic cell uptake by the phagocyte and involved in signaling events during the elimination of apoptotic cells. In addition, SR-F1 can trigger internalization of CRT, identified as an eat-me signal, but also able to modulate macrophage function.

## Data Availability Statement

The datasets generated for this study are available on request to the corresponding authors.

## Author Contributions

CW-P, NT, PF, and DH designed the study. CW-P, IB, SD, PT-D, NT, and PF performed the research. CW-P, NT, PF, PT-D, and DH analyzed the data. YD contributed key reagents. CW-P, NT, PF, and DH wrote the manuscript draft. All authors revised and approved the final version of the manuscript.

### Conflict of Interest

The authors declare that the research was conducted in the absence of any commercial or financial relationships that could be construed as a potential conflict of interest.

## Abbreviations

AcLDL: acetylated low density lipoprotein
CLR: collagen-like region of C1q
CRT: calreticulin
DC: dendritic cells
EGF: epidermal growth factor
GFP: green fluorescent protein
GR: globular region of C1q
LDL: low density lipoprotein
PMA: phorbol 12-myristate 13-acetate
PNGase F: N-glycosidase F
PS: phosphatidylserine
SPR: surface plasmon resonance
SREC: scavenger receptor expressed by endothelial cells
SR-F: scavenger receptor class F
TSA: thermal shift assay.

## References

[1] PrabhuDas MR Baldwin CL Bollyky PL Bowdish DME Drickamer K Febbraio M. A consensus definitive classification of scavenger receptors and their roles in health and disease. J Immunol. (2017) 198:3775–89. 10.4049/jimmunol.1700373 28483986 PMC5671342

[2] Adachi H Tsujimoto M Arai H Inoue K. Expression cloning of a novel scavenger receptor from human endothelial cells. J Biol Chem. (1997) 272:31217–20. 10.1074/jbc.272.50.31217 9395444

[3] Means TK Mylonakis E Tampakakis E Colvin RA Seung E Puckett L. Evolutionarily conserved recognition and innate immunity to fungal pathogens by the scavenger receptors SCARF1 and CD36. J Exp Med. (2009) 206:637–53. 10.1084/jem.20082109 19237602 PMC2699123

[4] Tamura Y Osuga J Adachi H Tozawa R Takanezawa Y Ohashi K. Scavenger receptor expressed by endothelial cells I (SREC-I) mediates the uptake of acetylated low density lipoproteins by macrophages stimulated with lipopolysaccharide. J Biol Chem. (2004) 279:30938–44. 10.1074/jbc.M313088200 15145948

[5] Pfistershammer K Klauser C Leitner J Stöckl J Majdic O Weichhart T. Identification of the scavenger receptors SREC-I, Cla-1 (SR-BI), and SR-AI as cellular receptors for Tamm-Horsfall protein. J Leukoc Biol. (2008) 83:131–8. 10.1189/jlb.0407231 17928461

[6] Nagase T Seki N Tanaka A Ishikawa K Nomura N. Prediction of the coding sequences of unidentified human genes. IV. The coding sequences of 40 new genes (KIAA0121-KIAA0160) deduced by analysis of cDNA clones from human cell line KG-1. DNA Res. (1995) 2:167–74. 10.1093/dnares/2.4.167 8590280

[7] Ishii J Adachi H Aoki J Koizumi H Tomita S Suzuki T. SREC-II, a new member of the scavenger receptor type F family, trans-interacts with SREC-I through its extracellular domain. J Biol Chem. (2002) 277:39696–702. 10.1074/jbc.M206140200 12154095

[8] Goldstein JL Ho YK Basu SK Brown MS. Binding site on macrophages that mediates uptake and degradation of acetylated low density lipoprotein, producing massive cholesterol deposition. Proc Natl Acad Sci USA. (1979) 76:333–7. 10.1073/pnas.76.1.333 218198 PMC382933

[9] Patten DA. SCARF1: a multifaceted, yet largely understudied, scavenger receptor. Inflamm Res. (2018) 67:627–32. 10.1007/s00011-018-1154-7 29725698 PMC6028831

[10] Adachi H Tsujimoto M. Characterization of the human gene encoding the scavenger receptor expressed by endothelial cell and its regulation by a novel transcription factor, endothelial zinc finger protein-2. J Biol Chem. (2002) 277:24014–21. 10.1074/jbc.M201854200 11978792

[11] Patten DA Kamarajah SK Rose JM Tickle J Shepherd EL Adams DH. SCARF-1 promotes adhesion of CD4+ T cells to human hepatic sinusoidal endothelium under conditions of shear stress. Sci Rep. (2017) 7:17600. 10.1038/s41598-017-17928-4 29242513 PMC5730566

[12] Jeannin P Bottazzi B Sironi M Doni A Rusnati M Presta M. Complexity and complementarity of outer membrane protein A recognition by cellular and humoral innate immunity receptors. Immunity. (2005) 22:551–60. 10.1016/j.immuni.2005.03.008 15894273

[13] Rechner C Kühlewein C Müller A Schild H Rudel T. Host glycoprotein Gp96 and scavenger receptor SREC interact with PorB of disseminating *Neisseria gonorrhoeae* in an epithelial invasion pathway. Cell Host Microbe. (2007) 2:393–403. 10.1016/j.chom.2007.11.002 18078691

[14] Baur S Rautenberg M Faulstich M Faulstich M Grau T Severin Y. A nasal epithelial receptor for *Staphylococcus aureus* WTA governs adhesion to epithelial cells and modulates nasal colonization. PLoS Pathog. (2014) 10:e1004089. 10.1371/journal.ppat.1004089 24788600 PMC4006915

[15] Beauvillain C Meloni F Sirard J-C Blanchard S Jarry U Scotet M. The scavenger receptors SRA-1 and SREC-I cooperate with TLR2 in the recognition of the hepatitis C virus non-structural protein 3 by dendritic cells. J Hepatol. (2010) 52:644–51. 10.1016/j.jhep.2009.11.031 20338659

[16] Thériault JR Adachi H Calderwood SK. Role of scavenger receptors in the binding and internalization of heat shock protein 70. J Immunol. (2006) 177:8604–11. 10.4049/jimmunol.177.12.8604 17142759

[17] Facciponte JG Wang X-Y Subjeck JR. Hsp110 and Grp170, members of the Hsp70 superfamily, bind to scavenger receptor-A and scavenger receptor expressed by endothelial cells-I. Eur J Immunol. (2007) 37:2268–79. 10.1002/eji.200737127 17615582

[18] Murshid A Gong J Calderwood SK. Heat shock protein 90 mediates efficient antigen cross presentation through the scavenger receptor expressed by endothelial cells-I. J Immunol. (2010) 185:2903–17. 10.4049/jimmunol.0903635 20686127 PMC4109054

[19] Murshid A Gong J Calderwood SK. Hsp90-peptide complexes stimulate antigen presentation through the class II pathway after binding scavenger receptor SREC-I. Immunobiology. (2014) 219:924–31. 10.1016/j.imbio.2014.08.001 25155057 PMC4886339

[20] Hölzl MA Hofer J Steinberger P Pfistershammer K Zlabinger GJ. Host antimicrobial proteins as endogenous immunomodulators. Immunol Lett. (2008) 119:4–11. 10.1016/j.imlet.2008.05.003 18573543

[21] Hölzl MA Hofer J Kovarik JJ Roggenbuck D Reinhold D Goihl A. The zymogen granule protein 2 (GP2) binds to scavenger receptor expressed on endothelial cells I (SREC-I). Cell Immunol. (2011) 267:88–93. 10.1016/j.cellimm.2010.12.001 21190681 PMC3040788

[22] Murshid A Borges TJ Calderwood SK. Emerging roles for scavenger receptor SREC-I in immunity. Cytokine. (2015) 75:256–60. 10.1016/j.cyto.2015.02.009 25767073 PMC4553087

[23] Ramirez-Ortiz ZG Pendergraft WF Prasad A Byrne MH Iram T Blanchette CJ. The scavenger receptor SCARF1 mediates the clearance of apoptotic cells and prevents autoimmunity. Nat Immunol. (2013) 14:917–26. 10.1038/ni.2670 23892722 PMC3752698

[24] Païdassi H Tacnet-Delorme P Garlatti V Darnault C Ghebrehiwet B Gaboriaud C. C1q binds phosphatidylserine and likely acts as a multiligand-bridging molecule in apoptotic cell recognition. J Immunol. (2008) 180:2329–38. 10.4049/jimmunol.180.4.2329 18250442 PMC2632962

[25] Zhou Z Hartwieg E Horvitz HR. CED-1 is a transmembrane receptor that mediates cell corpse engulfment in *C. elegans*. Cell. (2001) 104:43–56. 10.1016/s0092-8674(01)00190-8 11163239

[26] Kinchen JM Cabello J Klingele D Wong K Feichtinger R Schnabel H. Two pathways converge at CED-10 to mediate actin rearrangement and corpse removal in *C. elegans*. Nature. (2005) 434:93–9. 10.1038/nature03263 15744306

[27] Iram T Ramirez-Ortiz Z Byrne MH Coleman UA Kingery ND Means TK. Megf10 is a receptor for C1Q that mediates clearance of apoptotic cells by astrocytes. J Neurosci. (2016) 36:5185–92. 10.1523/JNEUROSCI.3850-15.2016 27170117 PMC4863057

[28] Païdassi H Tacnet-Delorme P Verneret M Gaboriaud C Houen G Duus K. Investigations on the C1q-calreticulin-phosphatidylserine interactions yield new insights into apoptotic cell recognition. J Mol Biol. (2011) 408:277–90. 10.1016/j.jmb.2011.02.029 21352829

[29] Verneret M Tacnet-Delorme P Osman R Awad R Grichine A Kleman J-P. Relative contribution of c1q and apoptotic cell-surface calreticulin to macrophage phagocytosis. J Innate Immun. (2014) 6:426–34. 10.1159/000358834 24557008 PMC6741593

[30] Malhotra R Willis AC Jensenius JC Jackson J Sim RB. Structure and homology of human C1q receptor (collectin receptor). Immunology. (1993) 78:341–8. 8478019 PMC1421832

[31] Kishore U Sontheimer RD Sastry KN Zaner KS Zappi EugeneG Hughes GRV. Release of calreticulin from neutrophils may alter C1q-mediated immune functions. Biochem J. (1997) 322:543–50. 10.1042/bj3220543 9065775 PMC1218224

[32] Kovacs H Campbell ID Strong P Johnson S Ward FJ Reid KB. Evidence that C1q binds specifically to CH2-like immunoglobulin gamma motifs present in the autoantigen calreticulin and interferes with complement activation. Biochemistry. (1998) 37:17865–74. 10.1021/bi973197p 9922153

[33] Moreau C Bally I Chouquet A Bottazzi B Ghebrehiwet B Gaboriaud C. Structural and functional characterization of a single-chain form of the recognition domain of complement protein C1q. Front Immunol. (2016) 7:79. 10.3389/fimmu.2016.00079 26973654 PMC4774423

[34] Berwin B Delneste Y Lovingood RV Post SR Pizzo SV. SREC-I, a type F scavenger receptor, is an endocytic receptor for calreticulin. J Biol Chem. (2004) 279:51250–7. 10.1074/jbc.M406202200 15371419

[35] Arlaud GJ Sim RB Duplaa AM Colomb MG. Differential elution of Clq, Clr and Cls from human Cl bound to immune aggregates. Use in the rapid purification of Cl subcomponents. Mol Immunol. (1979) 16:445–50. 10.1016/0161-5890(79)90069-5 40870

[36] Tacnet-Delorme P Chevallier S Arlaud GJ. Beta-amyloid fibrils activate the C1 complex of complement under physiological conditions: evidence for a binding site for A beta on the C1q globular regions. J Immunol. (2001) 167:6374–81. 10.4049/jimmunol.167.11.6374 11714802

[37] Tarentino AL Gómez CM Plummer TH. Deglycosylation of asparagine-linked glycans by peptide:N-glycosidase F. Biochemistry. (1985) 24:4665–71. 10.1021/bi00338a028 4063349

[38] Aude CA Lacroix MB Arlaud GJ Gagnon J Colomb MG. Differential accessibility of the carbohydrate moieties of Cls-Clr-Clr-Cls, the catalytic subunit of human Cl. Biochemistry. (1988) 27:8641–8. 10.1021/bi00423a020 3064815

[39] Laemmli UK. Cleavage of structural proteins during the assembly of the head of bacteriophage T4. Nature. (1970) 227:680–5. 10.1038/227680a0 5432063

[40] Osman R Tacnet-Delorme P Kleman J-P Millet A Frachet P. Calreticulin release at an early stage of death modulates the clearance by macrophages of apoptotic cells. Front Immunol. (2017) 8:1034. 10.3389/fimmu.2017.01034 28878781 PMC5572343

[41] Liu Y Annis DS Mosher DF. Interactions among the epidermal growth factor-like modules of thrombospondin-1. J Biol Chem. (2009) 284:22206–12. 10.1074/jbc.M109.026120 19531495 PMC2755945

[42] Sano M Korekane H Ohtsubo K Yamaguchi Y Kato M Shibukawa Y. N-glycans of SREC-I (scavenger receptor expressed by endothelial cells): essential role for ligand binding, trafficking and stability. Glycobiology. (2012) 22:714–24. 10.1093/glycob/cws010 22279061

[43] Fisher WR Hammond MG Warmke GL. Measurements of the molecular weight variability of plasma low density lipoproteins among normals and subjects with hyper- -lipoproteinemia. Demonstration of macromolecular heterogeneity. Biochemistry. (1972) 11:519–25. 10.1021/bi00754a006 4334903

[44] Crossley J Jones M Spragg SP Noble N Slack J Smethurst PR. Measurements of molecular weights of low-density lipoprotein (LDL) from homozygotes for familial hypercholesterolemia and controls. Biochem Med. (1981) 26:47–59. 10.1016/0006-2944(81)90029-6 7295303

[45] Crouse JR Parks JS Schey HM Kahl FR. Studies of low density lipoprotein molecular weight in human beings with coronary artery disease. J Lipid Res. (1985) 26:566–74. 4020295

[46] Reid KBM. Complement component C1q: historical perspective of a functionally versatile, and structurally unusual, serum protein. Front Immunol. (2018) 9:764. 10.3389/fimmu.2018.00764 29692784 PMC5902488

[47] Lebkowski JS McNally MA Okarma TB Lerch LB Inducible gene expression from multiple promotes by the tumor-promoting agent, PMA. Nucleic Acids Res. (1987) 15:9043–55. 3684579 10.1093/nar/15.21.9043 PMC306421

[48] Thielens NM Tedesco F Bohlson SS Gaboriaud C Tenner AJ. C1q: A fresh look upon an old molecule. Mol Immunol. (2017) 89:73–83. 10.1016/j.molimm.2017.05.025 28601358 PMC5582005

[49] Jin J Wang Y Ma Q Wang N Guo W Jin B. LAIR-1 activation inhibits inflammatory macrophage phenotype *in vitro*. Cell Immunol. (2018) 331:78–84. 10.1016/j.cellimm.2018.05.011 29887420

[50] Michée S Brignole-Baudouin F Riancho L Rostene W Baudouin C Labbé A. Effects of benzalkonium chloride on THP-1 differentiated macrophages *in vitro*. PLoS ONE. (2013) 8:e72459. 10.1371/journal.pone.0072459 23991114 PMC3747170

[51] Fox S Ryan KA Berger AH Petro K Das S Crowe SE. The role of C1q in recognition of apoptotic epithelial cells and inflammatory cytokine production by phagocytes during *Helicobacter pylori* infection. J Inflamm. (2015) 12:51. 10.1186/s12950-015-0098-8 26357509 PMC4563842

