# Supplementary material for: Molecular and Cellular Interactions of Scavenger Receptor SR-F1 With Complement C1q Provide Insights Into Its Role in the Clearance of Apoptotic Cells
Source: Front Immunol. 2020 Mar 31;11:544. doi: 10.3389/fimmu.2020.00544 (PMC7137648; doi:10.3389/fimmu.2020.00544)
Supplement: Supplementary file 1 [file Data_Sheet_1.pdf]

Supplementary Material

Molecular and cellular interactions of scavenger receptor SR-F1 with complement C1q provide insight into its role in the clearance of apoptotic cells

Catherine Wicker-Planquart, Samy Dufour, Pascale Tacnet-Delorme, Isabelle Bally, Yves Delneste, Philippe Frachet*, Dominique Housset, Nicole M Thielens*

*** Correspondence:** Nicole M. Thielens: nicole.thielens@ibs.fr
Philippe Frachet: philippe.frachet@ibs.fr

1 Supplementary Tables

Table S1 : Oligonucleotides

Name	Sequence
T7	TAATACGACTCACTATAGGG
Pac R2	CATCAGTGATGGTGATGATGATGATGATGCTTAATTAAAGTGTCCCGA CTGCCAGAGCC
BGHRev	TAGAAGGCACAGTCGAGG
PacF2	CACTTTAATTAAGCATCATCATCATCATCACCATCACTGATGACCTTG AATTTGGGGAGTGGG
221R	CATCAGTGATGGTGATGATGATGATGATGCTTAATTAACTGCTGGCAT TCGGGACCCCAC
353R	CATCAGTGATGGTGATGATGATGATGATGCTTAATTAAAGAGCCACAG TCTTCCCCAAAGGTAC
289F	CTGCGAGCCGGGCTGGCAAGGTACCCAGTGCCAGCAGC
289R	GCTGCTGGCACTGGGTACCTTGCCAGCCCGGCTCGCAG
382F	GGGGCCCAGCTGCCAAGCTTCCTGCCCAGCCG
382R	CGGCTGGGCAGGAAGCTTGGCAGCTGGGCCCC
393F	CCCAGCCGGTTTCCATGGTAACCAATGCTCAGTTCCTTGTGAATG
393R	CATTCACAAGGAACTGAGCATTGGTTACCATGGAAACCGGCTGGG
SRF1-stopF	CCAGGCCACCAGAACCCTAGTTTAAACGGCCGCTCGAGATGGAGAGC
SRF1-stopR	GCTCTCCATCTCGAGCGGCCGTTTAAACTAGGGTTCTGGTGGCCTGG

2 Supplementary Figures

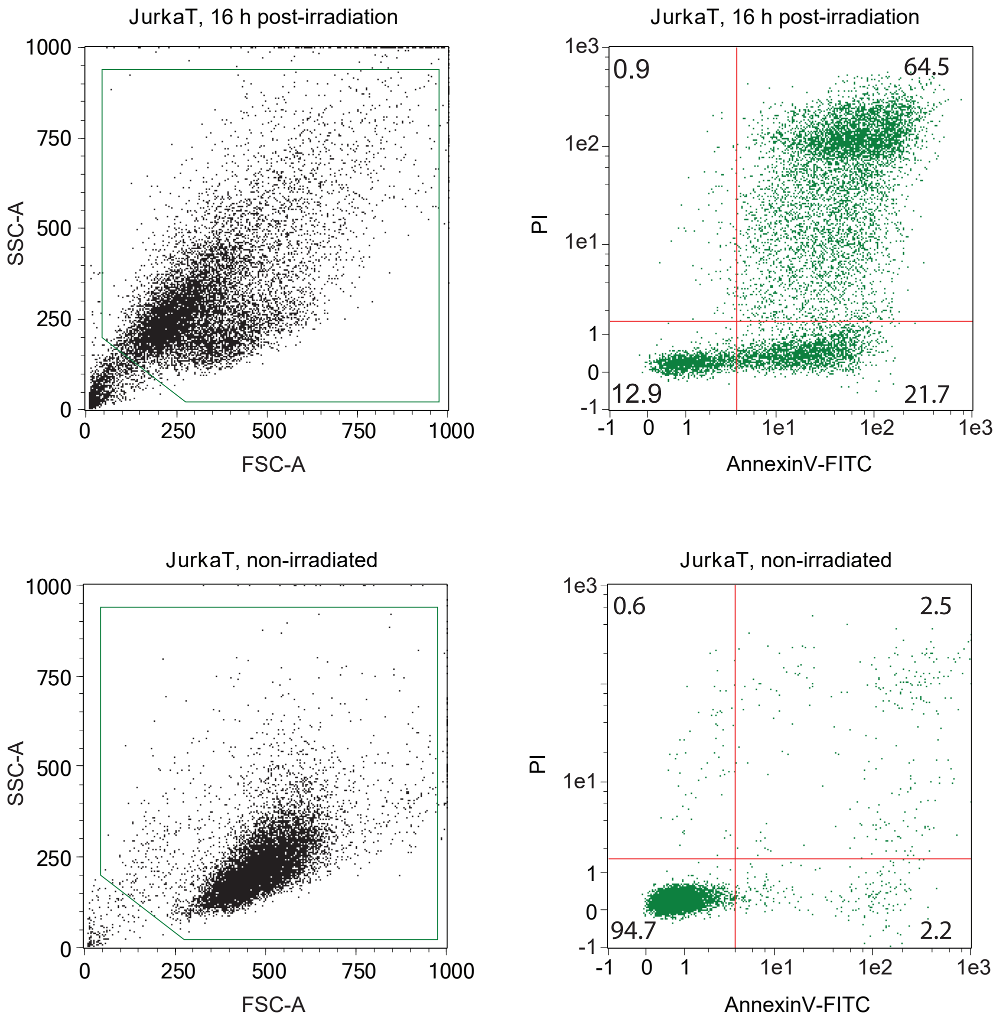

Figure S1: **Analysis of the apoptotic state of JurkaT cells used in the phagocytosis assays.** JurkaT cells were UVB-irradiated (312 nm) and then maintained for 16 h in cell culture. Apoptosis was assessed by flow cytometry using the Annexin V-FITC Kit (MACS MiltenyiBiotec) according to the manufacturer's instructions. FSC/SSC and AnnexinV/PI dot plots are shown for the irradiated and the control populations. The percent of AnnexinV-/PI-, AnnexinV-/PI+, AnnexinV+/PI- and AnnexinV+/PI+ in the selected population (green gates) are indicated. A representative experiment is shown.

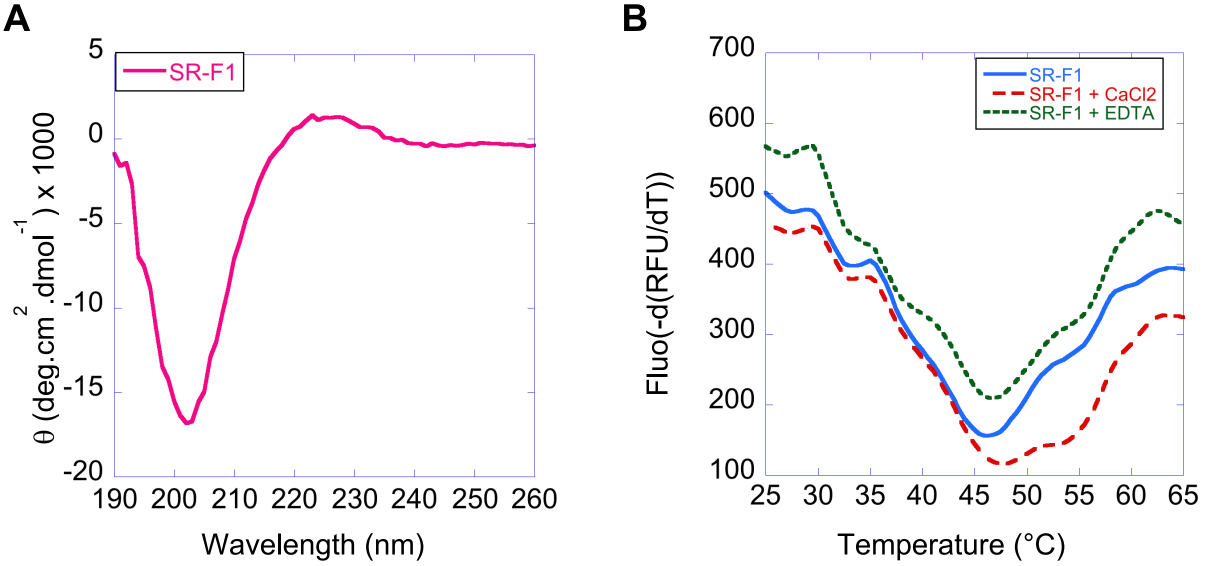

Figure S2. (**A**) CD spectra of SR-F1(20-421). Protein was dialyzed against 10 mM sodium phosphate, 150 mM NaF (pH 8) and used at a concentration of 8 µM. Ten spectra were acquired in the far-UV region (200-260 nm) at a scan rate of 20 nm/min. (**B**) Thermal shift analysis of SR-F1 stability in TBS buffer, in the presence of 2 mM $CaCl_2$ or 2 mM EDTA.

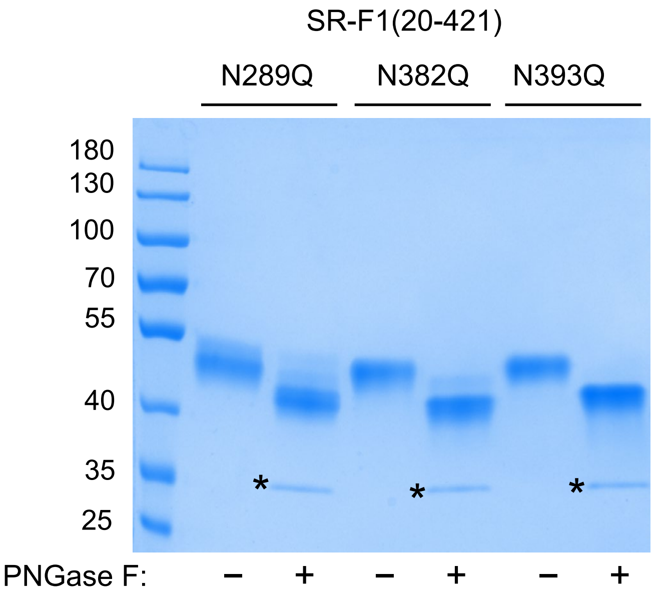

Figure S3. PNGase F deglycosylation of SR-F1(20-241) proteins. Proteins were digested by PNGase F as indicated in Materials and Methods and analysed by SDS-PAGE on a 12% acrylamide gel under unreduced conditions. Two µg of each protein were loaded on the gel. The molecular masses (kDa) of the marker are indicated. The band corresponding to PNGase F is indicated by a star.

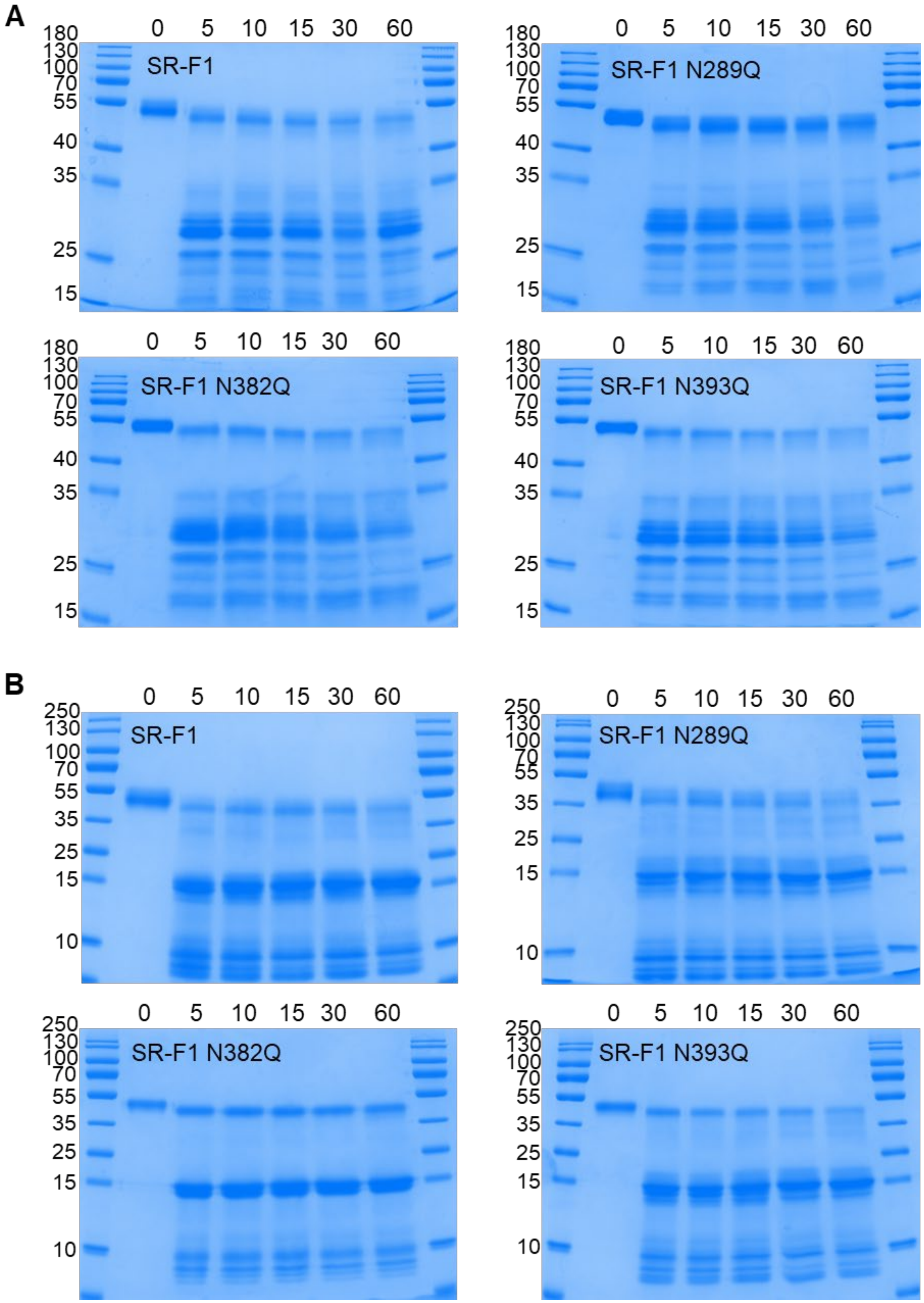

Figure S4. SDS-PAGE analysis of the proteolytic digestion of SR-F1(20-421) and its mutants lacking one N-glycosylation site. Limited proteolysis of the recombinant proteins (2 μg) was performed in TBS-2 mM $CaCl_2$ at 37 °C by addition of 0.1% trypsin (**A**) or chymotrypsin (**B**) for 0, 5, 10, 15, 30, and 60 min (indicated above the wells). The molecular masses of the protein mass markers (Fermentas) are indicated.

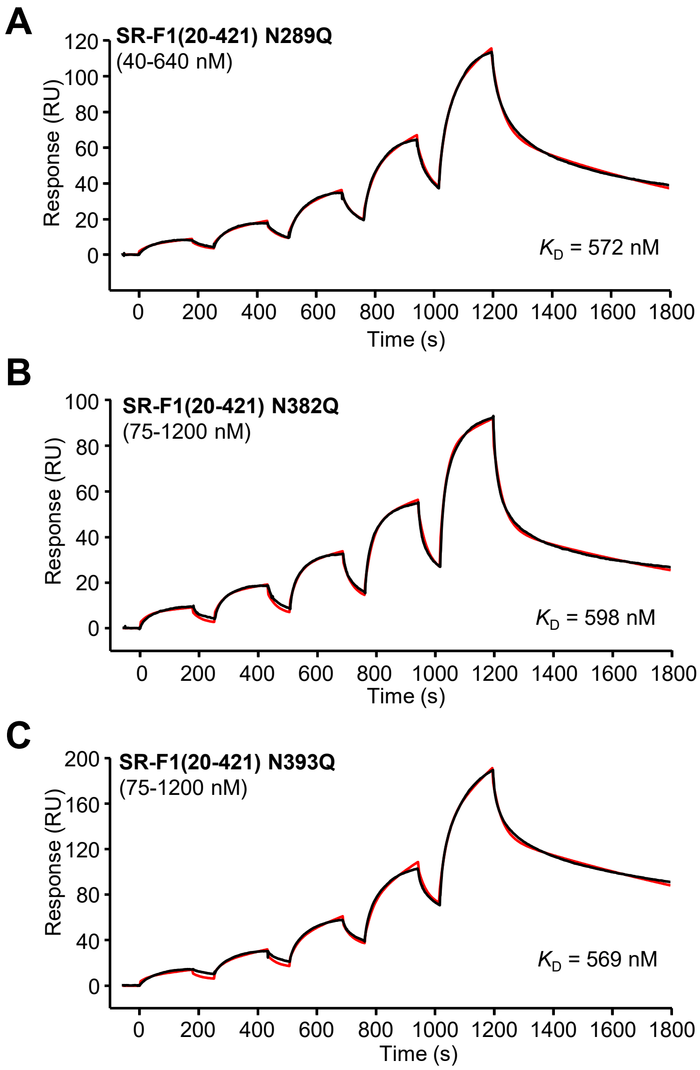

Figure S5. SPR analyses of the interaction of SR-F1 deglycosylation mutants with C1q. SR-F1(20-421) mutants N289Q (**A**), N382Q (**B**), N393Q (**C**) were serially diluted and injected at five increasing concentrations in single cycle kinetics mode over immobilized C1q (16,000 RU) in TBS-Ca-P at a flow rate of 20 µl/min. Fits are shown as red lines and apparent K_D values were obtained by global fitting of the data to a two-state reaction model. The data shown are representative of 2 separate experiments on different surfaces.

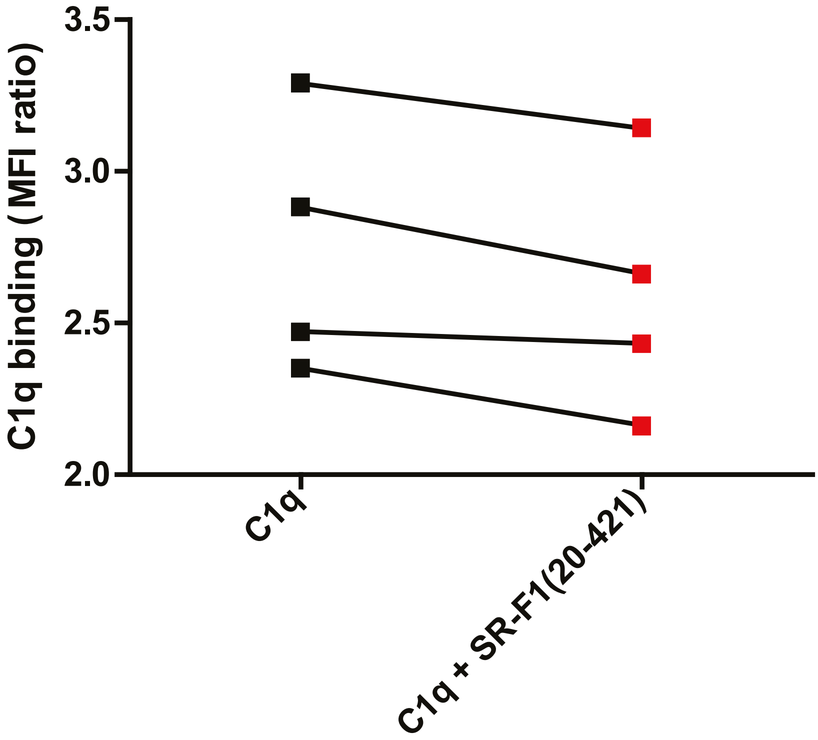

Figure S6. SR-F1(20-241) inhibits C1q binding to SR-F1 overexpressing THP-1 macrophages. C1q was preincubated for 30 min at 37°C without or with soluble SR-F1(20-241) protein at a 1:10 molar ratio (C1q:SR-F1) before its binding to SR-F1 overexpressing THP-1 macrophages. Incubation with PMA-treated THP-1 cells and C1q immunostaining were performed as described in Materials and Methods. Four independent experiments are shown. Decreased C1q binding is observed in the presence of soluble SR-F1 compared to controls. MFI ratio: MFI in the presence of C1q/MFI without C1q. MFI, median fluorescence intensity.

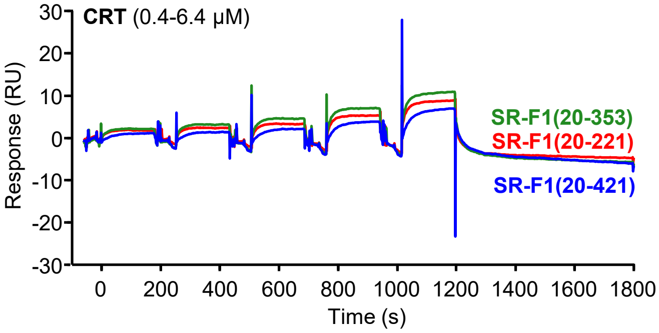

Figure S7. SPR analyses of the interaction of CRT with SR-F1(20-421), SR-F1(20-353) and SR-F1(20-421). CRT was serially diluted and injected in single cycle mode over covalently immobilized SR-F1(20-421) (4500 RU), SR-F1(20-353) (5800 RU) and SR-F1(20-221) (4700 RU) in TBS-Ca-P at a flow rate of 20 µl/min.
